# Prognostic value of parotid lymph node metastasis in patients with nasopharyngeal carcinoma receiving intensity-modulated radiotherapy

**DOI:** 10.1038/srep13919

**Published:** 2015-09-08

**Authors:** Yuan Zhang, Wen-Fei Li, Lei Chen, Yan-Ping Mao, Rui Guo, Fan Zhang, Hao Peng, Li-Zhi Liu, Li Li, Qing Liu, Jun Ma

**Affiliations:** 1 Department of Radiation Oncology, Sun Yat-sen University Cancer Center, State Key Laboratory of Oncology in South China, Collaborative Innovation Center for Cancer Medicine, 651 Dongfeng Road East, Guangzhou 510060, People’s Republic of China; 2 Imaging Diagnosis and Interventional Center, Sun Yat-sen University Cancer Center, State Key Laboratory of Oncology in South China, Collaborative Innovation Center for Cancer Medicine, 651 Dongfeng Road East, Guangzhou 510060, People’s Republic of China; 3 Department of Medical Statistics and Epidemiology, School of Public Health, Sun Yat–sen University, 74 Zhongshan Second Road, Guangzhou 510080, People’s Republic of China

## Abstract

The prognostic value and staging category of parotid lymph node (PLN) metastasis in nasopharyngeal carcinoma (NPC) remain unknown. We retrospectively reviewed MRI scans and medical records for 1811 NPC patients who received intensity-modulated radiotherapy. The diagnosis of PLN metastasis was mainly based on MRI follow-up. Twenty-five positive PLNs in 21/1811 patients were identified; the incidence of PLN metastasis was 1.2%. PLN metastasis was significantly associated with advanced N-category and stage. Ten of the 21 patients received irradiation of the involved PLNs; the PLN recurrence rate was significantly higher for patients who received no irradiation; thus only patients with irradiated PLN were included in prognostic analyses. PLN metastasis was associated with significantly poorer progression-free survival, overall survival and distant metastasis-free survival (DMFS), but not regional or local relapse-free survival, in univariate analysis. In multivariate analysis, PLN metastasis was also significantly associated with poor DMFS. PLN involvement had a significantly higher hazard ratio (HR) for distant failure than N2 disease and similar HR to N3 disease. In conclusion, PLN metastasis is rare in NPC and was associated with similarly poor DMFS as N3 disease. PLN metastasis should be suspected in advanced nodal disease, but diagnosed with care before administering aggressive treatment.

Nasopharyngeal carcinoma (NPC) is a malignant head and neck cancer especially common in Southern China, where the annual incidence is 15–25 cases per 100,000^1^. NPC has a high predilection for regional lymph node metastasis, up to 85% of cases present with lymphadenopathy at diagnosis^2^. The pattern of nodal metastasis in NPC follows an orderly spread down the neck with infrequent node skipping; the most commonly involved regions include the retropharyngeal space, levels II, III, IV and V, followed by level Ib and the supraclavicular nodes^2^,^3^,^4^,^5^,^6^,^7^.

The parotid lymph nodes (PLNs), classified as level VIII in the latest International Consensus Guidelines for nodal levels^8^, are also at risk of harboring metastasis from the nasopharynx. The occurrence of PLN metastasis has been reported in several studies and is approximately 1%^4^,^5^,^6^,^9^,^10^,^11^ To the best of our knowledge, the prognostic significance of and clinical staging system for PLN metastasis have not been investigated in NPC due the low incidence PLN involvement. Additionally, PLN metastasis is not included in the current International Union Against Cancer/American Joint Committee on Cancer (UICC/AJCC) staging system for NPC^12^, which results in varied classifications and treatment strategies for PLN metastasis at different centers.

Nowadays, intensity-modulated radiotherapy (IMRT) has replaced two-dimensional conventional radiotherapy as the main radiation technique for NPC. Compared with conventional radiotherapy, IMRT can spare the parotid glands from a high radiation dose, which significantly reduces the incidence of severe xerostomia^13^,^14^,^15^. However, several investigators recently reported recurrence of NPC in the parotid region after IMRT and inadequate irradiation of microscopic disease in the region of a spared parotid gland is considered to be one reason for such recurrences^16^,^17^,^18^,^19^,^20^. Thus the prognostic value and treatment of PLN metastasis in NPC has begun to attract more and more attention.

The diagnosis of PLN metastasis in NPC is mainly based on magnetic resonance imaging (MRI) and/or fine-needle aspiration cytology (FNAC). Compared to FNAC, MRI is a noninvasive and commonly used method for discrimination between benign and malignant parotid masses^21^,^22^. Moreover, several studies have indicated that imaging during follow-up may be a reliable method to determine the final diagnosis of lymph node involvement^23^,^24^,^25^. Thus, the aim of the present study was to determine the prognostic value of and staging category for PLN metastasis in a large cohort of patients with NPC treated with IMRT based on MRI follow-up.

## Methods

### Patients

From October 2009 to February 2012, 1811 patients with newly-diagnosed, non-distant metastatic, histologically-proven NPC treated with IMRT at our institution were retrospectively reviewed. All patients underwent a comprehensive pre-treatment evaluation, including a complete medical history, physical examination, hematology and biochemistry profiles, nasopharyngeal fiberoptic endoscopy, nasopharyngeal and neck MRI, chest radiography, abdominal sonography and whole body bone scan using 99mTc-methyldiphosphonate single photon emission computed tomography (SPECT). Additionally, 29.2% (528/1811) of the patients received a (18)F-fluorodeoxyglucose (18F-FDG) positron emission tomography CT (PET/CT) examination. All patients were restaged according to the 7^th^ edition of the UICC/AJCC staging system^12^. The clinicopathological characteristics of the patients are summarized in Table 1. This study was approved by the institutional review board at Sun Yat-sen University Cancer Center and carried out in accordance with the approved guidelines. All patients provided written informed consent for the treatment, publication of this report and all accompanying images.

### Imaging protocol

The region from the suprasellar cistern to the inferior margin at the sternal end of the clavicle was examined in each patient by MRI using a 1.5-T system (Signa CV/i; General Electric Healthcare, Chalfont St. Giles, United Kingdom) with a head-and-neck combined coil. Axial, coronal and sagittal T1-weighted fast spin-echo images (repetition time 500–600 ms, echo time 10–20 ms, 22 cm field of view) and axial T2-weighted fast spin-echo MR images (repetition time 4,000–6,000 ms, echo time 95–110 ms, 22 cm field of view) were obtained before intravenous injection of contrast material (0.1 mmol/kg gadopentetate dimeglumine; Magnevist, Schering, Berlin, Germany), then spin-echo T1-weighted axial and sagittal and spin-echo T1-weighted fat-suppressed coronal sequences (section thickness 5 mm, matrix size 512 × 512) were performed sequentially using the same parameters.

### Image assessment and diagnostic criteria

All MRI scans and clinical records were independently reviewed by two radiologists with over ten years’ experience each in head and neck cancer MRI; disagreements were resolved by consensus. Assessment of the PLNs included the subcutaneous pre-auricular nodes, superficial and deep intraparotid nodes and subparotid nodes^8^. The PLNs extend from the zygomatic arch and external auditory canal down to the mandible, from the subcutaneous tissue laterally to the styloid process medially, and from the posterior edge of the masseter and pterygoid muscles anteriorly to the anterior edge of the sternocleidomastoid muscle and posterior belly of the digastric muscle posteriorly^8^. All parotid nodules with a diameter ≥5 mm were recorded^26^,^27^.

The diagnosis of PLN metastasis was based on pretreatment pathology by FNAC and/or follow-up MRI. On follow-up MRI, a parotid lesion was considered to be a metastatic PLN if it achieved partial response (PR) or complete response (CR) according to the Response Evaluation Criteria in Solid Tumors^28^ three months after completion of radiotherapy or showed stable disease (SD) but increased in size during subsequent follow-up. A parotid lesion was considered negative if it showed SD at three months after completion of radiotherapy and did not progress during subsequent follow-up^25^.

### Treatment

The nasopharyngeal and neck tumor volumes of all patients were treated using IMRT for the entire course. Target volumes were delineated slice-by-slice on treatment planning CT scans using an individualized delineation protocol that complies with International Commission on Radiation Units and Measurements reports 50 and 62. The prescribed doses were 66–72 Gy in 28–33 fractions to the planning target volume (PTV) of the primary gross tumor volume (GTVnx), 64–70 Gy to the PTV of the GTV of the involved lymph nodes (GTVnd), 60–63 Gy to the PTV of the high-risk clinical target volume (CTV1), and 54–56 Gy to the PTV of the low-risk clinical target volume (CTV2). All targets were treated simultaneously using the simultaneous integrated boost technique. All patients were treated with one fraction daily over 5 days per week. During the study, institutional guidelines recommended only IMRT for stage I and concurrent chemoradiotherapy ± neoadjuvant/adjuvant chemotherapy for stage II to IVB. When possible, salvage treatments (intracavitary brachytherapy, surgery or chemotherapy) were provided in documented relapse or persistent disease.

### Follow-up and statistical analysis

Patient follow-up was measured from the first day of therapy to the day of last examination or death. Patients were examined at least every 3 months during the first 2 years, with follow-up examinations every 6 months for 3 years thereafter or until death. Progression–free survival (PFS) was calculated from Day 1 of treatment to the date of locoregional failure, distant failure or death from any cause, whichever occurred first. Overall survival (OS) was calculated from Day 1 of treatment to the date of last follow-up or death; distant metastasis–free survival (DMFS), local relapse–free survival (LRFS) and regional relapse–free survival (RRFS) to first distant metastasis, local recurrence or regional recurrence, respectively.

All statistical analyses were performed using SPSS v13.0 (Chicago, IL, USA). Categorical variables were compared using the Chi–square test (or Fisher’s exact test, if the expected number was less than five in at least one cell). Survival rates were calculated using the Kaplan-Meier method and compared using the log-rank test. Multivariate analyses with the Cox proportional hazards model^29^ were used to calculate hazard ratios (HRs), 95% confidence intervals (CIs) and test the independent significance of different factors by backward elimination of insignificant variables and included host factors (sex, age), therapeutic intervention (chemotherapy) and tumor factors (T-category, N-category) as covariates. Two-tailed *P*-values < 0.05 were considered statistically significant.

## Results

### Incidence of PLN metastasis

**Figure 1:**
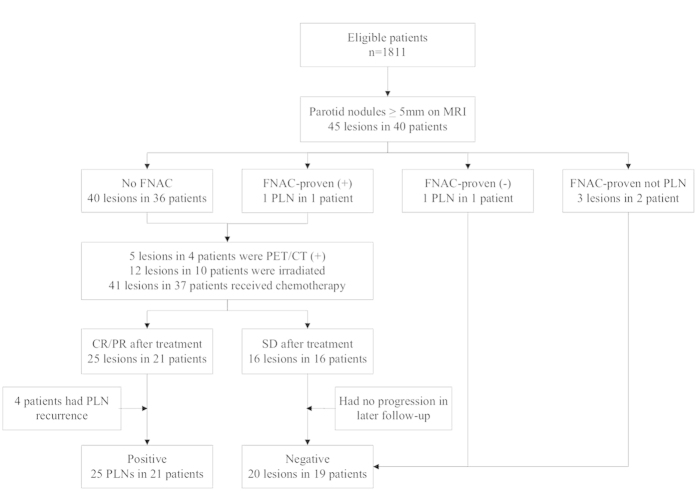
Flowchart illustrating the identification of positive parotid lymph nodes.

Among the 1811 patients with NPC, 45 parotid nodules with size ≥5 mm were identified in 40 patients in follow-up MRI imaging. Of these 40 patients, only four underwent pretreatment FNAC: one node was a metastatic PLN, one node was reactive lymphoid hyperplasia, one node was basal cell adenoma and the other node was fibrous connective tissue hyperplasia; the three patients without PLN metastases were excluded from further analyses. Among the remaining 37 patients, abnormal 18F-FDG uptake was observed in five lesions in four patients on PET/CT. The radiotherapy plans of these 37 patients were reviewed: 12 PLN lesions in ten patients had been included in the GTV or CTV receiving ≥54 Gy; all 37 patients also received chemotherapy. Three months after radiotherapy, 21 parotid lesions in 17/37 patients achieved CR, 4 lesions in 4 patients achieved PR, and 16 lesions in 16 patients achieved SD, while the primary tumor and cervical lymph nodes of these 37 patients all achieved CR or PR. Thus, 25 PLN metastases were identified in 21/1811 patients (Fig. 1); the incidence of PLN metastasis was 1.2%.

Among the 21 patients with PLN metastasis, 17 (81%) patients had a single involved PLN and four patients (19%) had two involved PLNs; 20 patients (95.2%) had unilateral PLN involvement and one patient (4.8%) had bilateral involvement. All 25 positive PLNs were located within the superficial lobe, separate from the primary tumor. The median minimal and maximal axial dimension values of the positive PLNs were 8.5 mm (range, 5.6–16.7 mm) and 11 mm (range, 5.8–17.6 mm), respectively. Two PLNs had central necrosis and no PLN showed extracapsular spread. Of these 21 patients, 19 (90.5%) had ipsilateral retropharyngeal lymphadenopathy, and also 19 (90.5%) had ipsilateral level II involvement.

The clinicopathological characteristics of the 21 patients with PLN metastasis are shown in Table 1. PLN metastasis was significantly associated with N-category (*P* < 0.01) and stage (*P* < 0.01), but not sex, age, histological type or T-category. Patients with more advanced N-category or stage group had a higher risk of PLN metastasis.

### Prognostic value of PLN metastasis

**Figure 2:**
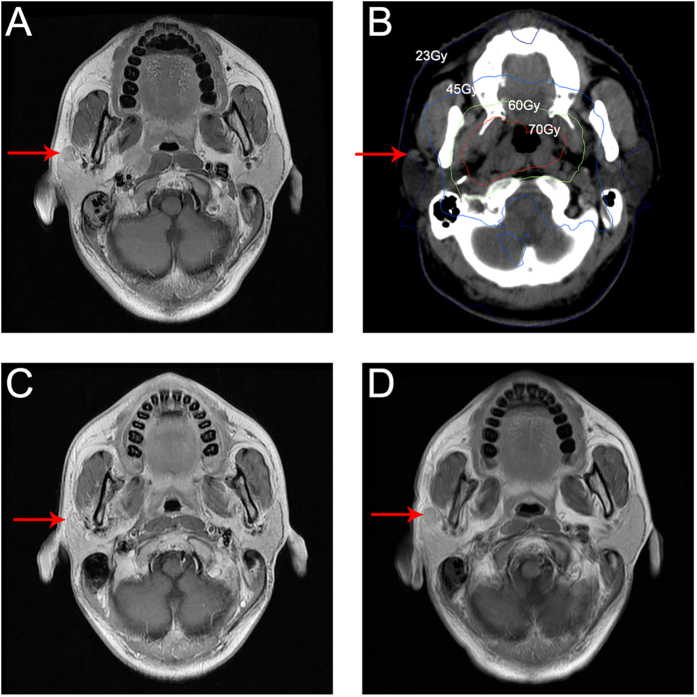
Imaging of parotid lymph node involvement in the right parotid gland in a 29-year-old male with NPC. (**A**) Pretreatment transverse T1-weighted spin-echo MRI image showing a 10 mm nonspecific hypointense nodule within the right parotid gland. (**B**) Isodose curves for intensity-modulated radiotherapy: red, 70 Gy; green, 60 Gy; light blue, 45 Gy; blue 23 Gy. (**C**) Transverse T1-weighted spin-echo MRI image 3 months after radiotherapy showing the lesion within the right parotid gland was controlled. (**D**) Transverse T1-weighted spin-echo MRI image 32 months after radiotherapy showing a 12 mm nonspecific hypointense nodule within the right parotid gland, which was pathologically confirmed as squamous carcinoma after surgery.

Median follow-up was 38.3 months (range, 1.3–60.2 months). The 3-year OS, PFS, DMFS, LRFS, and RRFS rates for the entire cohort were 94.2%, 84.7%, 90.7%, 95.7%, and 96.6%, respectively. Of the 21 patients with PLN metastasis, 10 patients (47.6%) experienced locoregional failure or distant metastasis and five (23.8%) died. Among the 10 patients with irradiated PLNs, there was no PLN recurrence during follow-up; however, four (36.4%) of the other 11 patients whose PLNs were not irradiated experienced PLN recurrence (*P* = 0.03). Typical pretreatment and recurrence MRI imaging and the isodose curves are shown in Fig. 2. The 11/21 patients who did not receive radical treatment for the PLNs were excluded from the prognostic analyses.

Univariate analysis of the remaining 1800 patients showed PLN metastasis was significantly associated with poorer PFS (HR, 3.27; 95% CI, 1.22–8.78; *P* = 0.02), OS (HR, 5.20; 95% CI, 1.65–16.37; *P* < 0.01) and DMFS (HR, 5.54; 95% CI, 2.05–14.96; *P* < 0.01), but not RRFS (*P* = 0.21) or LRFS (*P* = 0.68). Multivariate analysis was performed to adjust for various prognostic factors, including age, sex, T-category, N-category and chemotherapy. PLN metastasis was associated with significantly poorer DMFS (HR, 3.15; 95% CI, 1.16–8.60; *P* = 0.03) but not PFS (P = 0.20), OS (*P* = 0.13) or RRFS (*P* = 0.33; Table 2).

### Staging category for PLN metastasis

**Figure 3:**
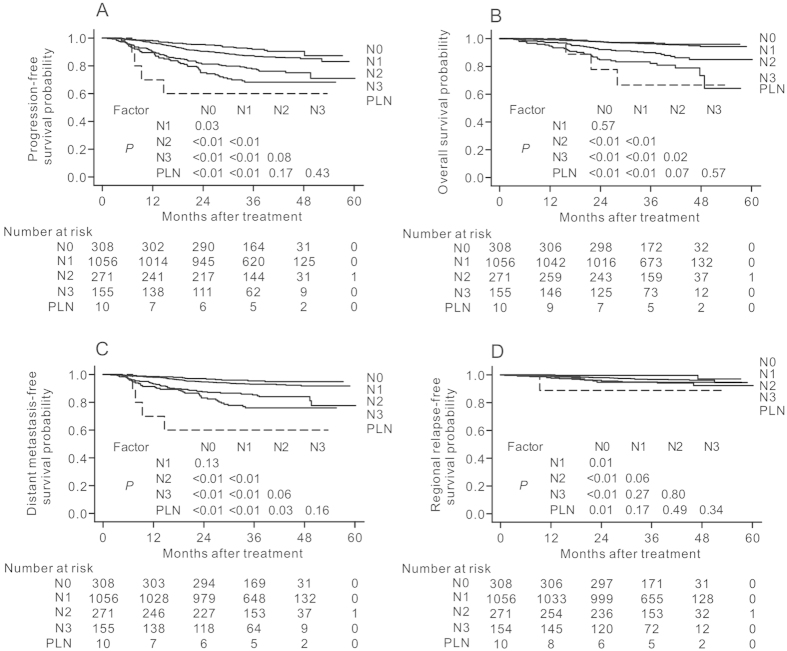
(**A**) Progression-free survival, (**B**) overall survival, (**C**) distant metastasis-free survival and (**D**) regional relapse-free survival curves for patients with NPC stratified by N-category (as defined by the 7th UICC/AJCC system) and parotid lymph node (PLN) involvement. The 11 patients whose involved PLNs were not irradiated were excluded.

To evaluate the relative severity of PLN metastasis, the 1800 patients were classified into five groups according to lymph node involvement: N0 disease (*n* = 308), N1 disease (n = 1056), N2 disease (*n* = 271), N3 disease (*n* = 155), and PLN metastasis (*n* = 10). The PLN metastasis group had the poorest PFS, OS, DMFS and RRFS rates of the five groups (Fig. 3). Compared to patients with N2 disease (HR = 1), patients with PLN metastasis had a significantly higher HR for distant failure (HR, 3.13; 95% CI, 1.12–8.73; *P* = 0.03; Table 3) adjusted for sex, age, chemotherapy and T-category. However, no significant difference in the HRs for progression or death (*P* = 0.45), death (*P* = 0.51), distant failure (*P* = 0.17) or regional failure (*P* = 0.18) were found between patients with PLN metastasis and patients with N3 disease.

## Discussion

To the best of our knowledge, this is the first study to investigate the prognostic value of PLN metastasis in a large cohort of patients with NPC treated with IMRT. PLN metastasis was rare (incidence of only 1.2%) but was significantly associated with advanced N-category and stage group. Moreover, PLN metastasis was an independent prognosticator for unfavorable DMFS, with a similar HR for distant failure as N3 disease.

The diagnosis of PLN metastasis in this study was mainly based on follow-up MRI rather than FNAC or radiologic criteria^26^ for several reasons. Firstly, NPC is mainly staged by MRI and treated with radiotherapy. When pathologic confirmation of the primary tumor is available, FNAC is seldom performed as an invasive examination in patients with parotid lesions^11^. Secondly, the PLNs are not routinely removed during dissection of the neck, thus the radiologic criteria based on clinicopathologic analysis of the neck lymph nodes in dissection specimens cannot be applied to the PLNs^26^, and radiologic criteria for PLN metastasis have not yet been defined. Thirdly, several studies have indicated that follow-up imaging may be a credible method for determining the final lymph node diagnosis, as malignant lymph nodes usually resolve after radiotherapy and/or chemotherapy or progress by the time of follow-up, whereas benign lesions often remain stable in size or remain negative during follow-up^23^,^24^,^25^. Although only 47.6% (10/21) of patients with positive PLN(s) were irradiated in this study, all the 21 patients received chemotherapy. Besides being radiosensitive, NPC is also sensitive to chemotherapy and platinum-containing regimens have high response rates of 72–100%^30^. Thus PLN metastasis can be presumed to have existed before treatment if the nodes achieved PR or CR after radiotherapy and/or chemotherapy^11^.

The PLNs receive efferent lymphatic drainage from the frontal and temporal skin, eyelids, conjunctiva, auricle, external acoustic meatus, tympanum, nasal cavities, root of the nose, nasopharynx and Eustachian tubes^8^. PLN metastasis is rare in NPC and a meta-analysis of 13 studies of 2920 cases of NPC staged via MRI showed that the PLNs had a low risk of metastasis (incidence of about 1% in all patients) and classified the PLNs as the third echelon of draining nodes in NPC^2^. In the current study, the incidence of PLN metastasis was 1.2% (21/1811), which is similar to the rates reported in former studies^4^,^5^,^6^,^9^,^10^. Of the 21 patients with PLN metastasis, 19 (90.5%) had ipsilateral retropharyngeal lymphadenopathy and also 19 (90.5%) had ipsilateral level II involvement, suggesting that the tumor may spread to the PLNs via the retropharyngeal lymph nodes or from enlarged superior cervical nodes in a retrograde fashion^11^,^20^. Thus, suspicion of PLN metastasis should be raised in patients with advanced nodal disease on pretreatment imaging.

As it has such a low incidence, the prognostic significance of PLN metastasis has not yet been reported in NPC and the PLNs are not included in the UICC/AJCC TNM staging system. In clinical practice, PLN involvement is often classified as N3 or M1 disease. In this study, PLN metastasis was significantly associated with advanced N-category and stage group, but not T-category. To better evaluate the prognostic value of PLN involvement, only the ten patients with irradiated PLN were included in the prognostic analyses. PLN metastasis was associated with significantly poorer PFS, OS and DMFS in univariate analysis and poorer DMFS independently of N-category in multivariate analysis. Moreover, the PLN group had a significantly higher HR for distant failure than patients with N2 disease, but similar HR as N3 disease. Although the number of patients with PLN metastasis was small and whether inclusion of the PLNs significantly affects N-categorization remains to be assessed, our data suggested that patients with PLN involvement had a higher risk of distant metastasis, and should receive more aggressive treatment in a similar manner to N3 disease.

In clinical practice, PLN metastasis is often neglected and does not receive a radical radiation dose, especially during parotid-gland-sparing IMRT^16^,^17^,^18^,^19^,^20^. In the study by Cannon and Lee^16^, two patients with small (5–8 mm) nonspecific PLNs that showed no hypermetabolic activity on pretreatment PET/CT suffered PLN recurrence after parotid-gland-sparing IMRT. In this study, only half of the positive PLNs were irradiated within the GTV or CTV, and the PLN recurrence rate was significantly higher for patients who received irradiation of involved PLN than those whose involved PLNs did not received irradiation (36.4% vs. 0%, *P* = 0.03). Our results indicated that radical radiotherapy was an effective method to treat PLN metastasis. However, considering that only 21/40 (52.5%) patients with pretreatment parotid lesions were eventually diagnosed with PLN metastasis during follow-up, we recommend FNAC and/or PET/CT should be performed in patients with suspicious PLNs before administering a radical or prophylactic dose in order to avoid overtreatment and the associated toxicities.

It should be stressed that this study was not a radiologic-histopathologic correlation study. The diagnosis of positive PLNs was mainly based on the findings of follow-up MRI imaging, rather than pathology, thus there might be false-negative and false-positive diagnoses. Secondly, only the ten patients with irradiated PLNs were included in the prognostic analyses. Thirdly, among the 11 patients whose involved PLNs were not irradiated, only four patients experienced PLN recurrence during follow-up; this could be due to the combined use of chemotherapy, the effect of the marginal dose around the PTV, the relatively short follow-up or false-positive diagnoses. Thus, these results need to be further verified in a large cohort pathology-based study.

In conclusion, this study confirms the low incidence of PLN metastasis in NPC, and provides the first evidence that PLN metastasis is associated with advanced N-category and poorer survival, similarly to N3 disease. PLN metastasis should be diagnosed with care in patients with advanced disease at pretreatment evaluation before prescribing aggressive treatment.

## Additional Information

**How to cite this article**: Zhang, Y. *et al.* Prognostic value of parotid lymph node metastasis in patients with nasopharyngeal carcinoma receiving intensity-modulated radiotherapy. *Sci. Rep.* **5**, 13919; doi: 10.1038/srep13919 (2015).

## Figures and Tables

**Table 1:** Clinicopathological characteristics of 1811 patients with NPC stratified by the presence or absence of parotid lymph node metastasis.

Characteristic	No. of 1790 patients (%) without PLN	No. of 21 patients (%) with PLN	*P*
**Sex**			0.24
Male	1333 (74.5%)	18 (85.7%)	
Female	457 (25.5%)	3 (14.3%)	
**Age (years)**			0.07
≤45	948 (53%)	7 (33.3%)	
>45	842 (47%)	14 (66.7%)	
**Histological type**			0.73
WHO type I	10 (0.6%)	0 (0%)	
WHO type II/III	1780 (99.4%)	21 (100%)	
**Chemotherapy**			0.06
No	259 (14.4%)	0 (0%)	
Yes	1531 (85.6%)	21 (100%)	
**T-category**			0.62
T1	323 (18%)	3 (14.3%)	
T2	278 (15.5%)	5 (23.8%)	
T3	857 (47.9%)	8 (38.1%)	
T4	332 (18.5%)	5 (23.8%)	
**N-category**			<0.01
N0	308 (17.2%)	0 (0%)	
N1	1056 (59%)	6 (28.6%)	
N2	271 (15.1%)	5 (23.8%)	
N3a	30 (1.7%)	2 (9.5%)	
N3b	125 (7%)	8 (38.1%)	
**Stage-group**			<0.01
I	99 (5.5%)	0 (0%)	
II	381 (21.3%)	1 (4.8%)	
III	851 (47.5%)	6 (28.6%)	
IVa	304 (17%)	4 (19%)	
IVb	155 (8.7%)	10 (47.6%)	

**Table 2:** Summary of multivariate analysis of different prognostic factors in 1800 patients with NPC.

Endpoint	Factor	HR	95% CI	*P*
Progression	Age > vs. ≤45 yrs	1.25	0.99–1.58	0.06
or death	Sex female vs. male	1.00	0.76–1.31	0.99
	Chemotherapy yes vs. no	0.94	0.63–1.41	0.76
	AJCC T-category T_3–4_ vs. T_1–2_	1.71	1.30–2.24	<0.01
	AJCC N-category N_2–3_ vs. N_0–1_	2.19	1.72–2.78	<0.01
	PLN metastasis yes vs. no	1.92	0.71–5.21	0.20
Death	Age > vs. ≤45 yrs	1.48	1.03–2.12	0.04
	Sex female vs. male	0.85	0.55–1.31	0.46
	Chemotherapy yes vs. no	0.70	0.37–1.31	0.26
	AJCC T-category T_3–4_ vs. T_1–2_	2.77	1.74–4.42	<0.01
	AJCC N-category N_2–3_ vs. N_0–1_	3.58	2.48–5.17	<0.01
	PLN metastasis yes vs. no	2.48	0.78–7.91	0.13
Distant failure	Age > vs. ≤45 yrs	1.09	0.81–1.48	0.57
	Sex female vs. male	0.75	0.51–1.09	0.13
	Chemotherapy yes vs. no	1.14	0.64–2.04	0.65
	AJCC T-category T_3–4_ vs. T_1–2_	1.78	1.25–2.52	<0.01
	AJCC N-category N_2–3_ vs. N_0–1_	2.74	2.01–3.73	<0.01
	PLN metastasis yes vs. no	3.15	1.16–8.60	0.03
Regional	Age >vs. ≤45 yrs	0.87	0.52–1.46	0.60
failure	Sex female vs. male	1.70	1.01–2.87	0.05
	Chemotherapy yes vs. no	0.80	0.36–1.80	0.59
	AJCC T-category T_3–4_ vs. T_1–2_	1.16	0.67–2.01	0.59
	AJCC N-category N_2–3_ vs. N_0–1_	1.92	1.15–3.19	0.01
	PLN metastasis yes vs. no	2.72	0.37–20.00	0.33

Abbreviations: HR = hazard ratio; CI = confidence interval; PLN = parotid lymph nodes. The 11 patients whose involved parotid lymph nodes were not irradiated were excluded from this analysis.

**Table 3:** Hazard consistency for different nodal subgroups in 1800 patients with NPC.

Endpoint	Category	HR	95% CI	*P*
Progression	N0	0.36	0.23–0.57	<0.01
or death	N1	0.54	0.40–0.73	<0.01
	N2	1		
	N3	1.48	1.01–2.16	0.04
	PLN metastasis	1.89	0.68–5.20	0.22
Death	N0	0.32	0.16–0.66	<0.01
	N1	0.34	0.22–0.54	<0.01
	N2	1		
	N3	1.98	1.20–3.26	<0.01
	PLN metastasis	2.57	0.78–8.46	0.12
Distant failure	N0	0.30	0.16–0.55	<0.01
	N1	0.43	0.30–0.63	<0.01
	N2	1		
	N3	1.61	1.02–2.53	0.04
	PLN metastasis	3.13	1.12–8.73	0.03
Regional failure	N0	0.11	0.03–0.48	<0.01
	N1	0.55	0.30–1.01	0.05
	N2	1		
	N3	0.85	0.35–2.10	0.73
	PLN metastasis	2.15	0.28–16.33	0.46

Abbreviations: HR = hazard ratio; CI = confidence interval; PLN = parotid lymph node. Adjusted for age (> vs. ≤45 yrs), sex (female vs. male), T-category (T_3–4_ vs. T_1–2_) and chemotherapy (yes vs. no). The 11 patients whose involved parotid lymph nodes were not irradiated were excluded from this analysis.
